# Snail mediates invasion through uPA/uPAR and the MAPK signaling pathway in prostate cancer cells

**DOI:** 10.3892/ol.2013.1635

**Published:** 2013-10-18

**Authors:** DIANDRA D. RANDLE, SHINEKA CLARKE, VERONICA HENDERSON, VALERIE A. ODERO-MARAH

**Affiliations:** Department of Biological Sciences, Center for Cancer Research and Therapeutic Development, Clark Atlanta University, Atlanta, GA 30314, USA

**Keywords:** Snail, invasion, urokinase plasminogen activator, urokinase plasminogen activator receptor, mitogen-activated protein kinase, prostate cancer

## Abstract

Epithelial-mesenchymal transition (EMT) is a process by which cancer cells acquire mesenchymal properties, such as induction of vimentin, while epithelial-associated genes like E-cadherin are lost. This enables cells to be more metastatic. Factors that are able to induce EMT include growth factors such as transforming growth factor-β (TGF-β) and epidermal growth factor, and transcription factors such as Snail. Snail-induced EMT promotes migration and invasion and we hypothesized that this may be mediated by the activity of urokinase-type plasminogen activator (uPA) and its receptor (uPAR). LNCaP, 22Rv1 and ARCaP human prostate cancer (CaP) cells stably transfected with empty vector control (Neo) or constitutively active Snail exhibited increased cell invasion. Superarray analysis revealed an upregulation in uPA and uPAR RNA expression in Snail-transfected ARCaP cells compared with that of a Neo control. In addition, the protein expression levels of Snail, uPA and uPAR were measured by western blot analysis which showed that overexpression of Snail increased uPA and uPAR protein levels. The activity of uPA in conditioned media was measured using an ELISA which revealed that uPA activity was elevated in LNCaP, 22Rv1 and ARCaP cells overexpressing Snail. Additionally, transient silencing of uPAR in ARCaP cells overexpressing Snail using short interfering RNA resulted in abrogation of Snail-mediated invasion. Snail overexpression was associated with increased extracellular-signal-regulated kinase activity, and antagonism of this activity with mitogen-activated protein (MAPK) inhibitor, UO126, inhibited cell invasion and decreased uPA activity. Therefore, Snail-mediated cell invasion in human CaP cells may occur via the regulation of uPA/uPAR and the MAPK signaling pathway.

## Introduction

Prostate cancer (CaP) is the most commonly diagnosed malignancy in the United States, with the majority of cases occurring in males over the age of 55 (1). In 2012, ~241,740 new cases of CaP were predicted to be diagnosed, with ~28,170 men succumbing to CaP, in the United States alone (1). Tumors that are detected early via testing serum prostate-specific antigen levels or digital rectal examination may be effectively treated by prostatectomy or radiation therapy (2). Approximately 30% of treated patients suffer relapse and progress to hormone refractory prostate cancer (HRPC), which no longer responds to androgen ablation, whereas early CaP growth is androgen-dependent. At that stage, there is no curative therapy available for metastatic CaP (3,4). Metastasis is a complex process by which cancer cells leave the primary tumor and migrate to a secondary site where they recolonize. It consists of multiple steps that are interconnected, including invasion, migration, intravasation, extravasation and recolonization (5,6). The shortcomings of treatment for such highly invasive and metastatic disease have led to several investigations of various molecular targets that directly affect invasion and metastasis with the aim of developing safe and effective treatments.

Numerous studies suggest that epithelial-mesenchymal transition (EMT) may be an important step leading to cancer metastasis (7–9). A notable mechanism by which E-cadherin is downregulated in EMT is transcriptional repression by Snail (10,11). Induction of Snail expression has been noted in a number EMT processes that have been studied (11–13). Additionally, increases in signaling in survival pathways such as mitogen-activated protein kinase (MAPK) is associated with increased Snail expression (14). Snail is composed of two interacting domains (12,15,16); the C-terminal domain is responsible for binding to DNA sequences with a 5′-CAGGTG-3′ core, while the N-terminal is required for transcriptional repression (16,17). Overexpression of Snail is sufficient to induce EMT and is associated with highly invasive tumors in mice and humans (18).

In order for tumors to colonize to a secondary site, they must invade the extracellular matrix (ECM) (5,6). Several proteolytic enzymes are involved in this process of degradation. Among these enzymes is the plasminogen activation (PA) system which leads to activation of matrix metalloproteases (MMPs) (19,20). The members of the PA system include urokinase-type plasminogen activator (uPA), plasminogen activator inhibitors (PAIs) and the uPA receptor (uPAR) (19,20). uPA, when bound to its cellular receptor uPAR, efficiently converts plasminogen into the broad-spectrum serine protease plasmin; its action on plasminogen is controlled by the serine protease inhibitors PAI-1 and PAI-2 (13–15). uPA catalyzes the activation of plasminogen into plasmin by cleaving the arginine-valine bond. In turn, plasmin facilitates the release of several proteolytic enzymes, including gelatinase and fibronectin (19–21).

It has been well established that uPA and uPAR, both members of the PA system, are involved in cancer invasion and metastases (19–23). It has been shown that plasma levels of uPA and uPAR are higher in males with CaP compared with healthy controls and significantly declined after prostate removal (24). Under normal conditions, uPAR is considered to have fairly limited tissue expression (25). Studies using mice and human clinical samples have identified conditions in which uPAR expression is induced (25,26). uPAR is induced during ECM remodeling, stress, injury and inflammation, and is highly expressed during tissue reorganization and inflammation, as well as in virtually all human cancers (19,21,25). Furthermore, it has been shown that uPAR is under an extracellular-signal-regulated kinase (ERK)-dependent mechanism and blocking uPAR’s activity leads to inhibition of motility in hepatocellular carcinoma (27). In human gastric cancer, studies have demonstrated that epidermal growth factor (EGF) stimulates uPAR expression via the ERK pathway, sequentially increasing cell invasion (28).

Several studies have shown that Snail mediates invasion through MMP activation (29–31); however, there are few studies that link Snail and uPA to cancer progression. One study indicated that silencing uPA expression in MDA-MB-231 breast cancer cells decreased expression of vimentin and Snail, and induced changes in morphology characteristic of epithelial cells (32). These results demonstrate that uPAR-initiated cell signaling may be targeted to reverse EMT in cancer (32). Another study suggested that when Snail is blocked in the invasive breast cancer cell-line MDA-MB-231, there is a decrease in the expression of PAI-1 and uPA transcripts and reduced migration (33).

Previously, we have stably overexpressed Snail in LNCaP and ARCaP CaP cell lines and shown that Snail led to EMT associated with decreased/relocalized E-cadherin, increased vimentin and increased migration (34–37). In this study, we investigated the molecular mechanisms of Snail-mediated cell invasion. We propose that Snail increases invasion via uPA/uPAR signaling. The results showed that Snail overexpression led to an increase in cell invasion, which was antagonized by uPAR silencing. Snail also increased the levels of uPA and uPAR protein, as well as uPA and ERK activities. Furthermore, the inhibition of MAPK activity decreased uPA activity and cell invasion. Our results show, for the first time, a link between Snail, MAPK and uPA/uPAR in CaP. This demonstrates that Snail regulates cell invasion via uPA-uPAR activites, possibly through the MAPK pathway.

## Materials and methods

### Reagents and antibodies

RPMI-1640 medium and penicillin/streptomycin were purchased from VWR International, Inc. (West Chester, PA, USA). The protease inhibitor cocktail was obtained from Roche Molecular Biochemicals (Indianapolis, IN, USA), while G418 and anti-human actin antibodies were purchased from Sigma-Aldrich, Inc. (St. Louis, MO, USA), and rabbit polyclonal anti-human Snail antibody and rabbit anti-phospho-ERK1/2 (p-ERK) were obtained from Cell Signaling Technology, Inc. (Danvers, MA, USA). Rabbit polyclonal anti-uPA, anti-uPAR and anti-total-ERK1/2 were purchased from Santa Cruz Biotechnology, Inc. (Santa Cruz, CA, USA). Horseradish peroxidase-conjugated sheep anti-mouse, sheep anti-rabbit and the ECL Prime or ECL Plus chemiluminescent reagents were obtained from GE Healthcare Life Sciences (Little Chalfont, UK. Fetal bovine serum (FBS) and dextran-coated charcoal-treated FBS (DCC-FBS) were supplied by HyClone (South Logan, UT, USA). Control and Snail short interfering RNA (siRNA) constructs were purchased from Dharmacon, Inc. (Lafayette, CO, USA), and UO126 was purchased from Sigma-Aldrich, Inc. The uPA Activity Assay kit was obtained from Millipore (Billerica, MA, USA) and Matrigel was purchased from BD Biosciences (Bedford, MA, USA).

### Cell culture

Human CaP cell line ARCaP (Cedar Sinai Medical Center, Los Angeles, CA, USA) stably transfected with constitutively active Snail cDNA (ARCaP Snail representing an aggressive cell line) or an empty vector Neo (ARCaP Neo representing the less aggressive cell line), as well as LNCaP cells overexpressing Snail, have been previously described as representing an EMT model and were utilized in these experiments (34–37). The 22Rv1 cells overexpressing Snail utilized in the present experiments were previously generated (35). The LNCaP human CaP cell line was obtained from American Type Culture Collection (Manassas, VA, USA) and maintained in RPMI-1640 (Corning Cellgro, Manassas, VA, USA), supplemented with 10% FBS, 1% non-essential amino acids and 1% antibiotics at 37°C in 5% CO_2_. The Snail-transfected cells were maintained in RPMI-1640 supplemented with 10% FBS, 1% non-essential amino acids and 1% antibiotics plus 400 μg/ml G418. All cells were maintained at 70–80% confluence.

### Western blot analysis

Cells were cultured to 85–90% confluency; subsequently, cells were washed with phosphate-buffered saline and harvested in modified RIPA buffer (50 mM Tris, pH 8.0; 150 mM NaCl; 0.02% NaN_3_; 0.1% sodium dodecyl sulfate; 1% NP-40; 0.5% sodium deoxycholate) containing 1.5X protease inhibitor cocktail, 1 mM phenylmethylsufonyl fluoride and 1 mM sodium orthovanadate. Protein concentrations were calculated using the bicinchoninic acid protein assay (Pierce, Rockford, IL, USA). Equal concentrations of whole cell protein were separated on a 10% SDS-polyacrylamide gel electrophoresis gel and transferred to a nitrocellulose membrane. Non-specific antibody binding sites were blocked using 3 or 5% non-fat dry milk and Tris-buffered saline and Tween-20 (TBST), and washed with TBST. Membranes were incubated with primary antibodies in 3% bovine serum albumin-TBST (p-ERK and Snail), or 5% non-fat dry milk and TBST (uPA, uPAR, ERK1/2 and β-actin) overnight at 4°C. Membranes were washed in TBST and incubated with HRP-conjugated sheep anti-rabbit (Snail, uPA, uPAR and p-ERK) or anti-mouse (actin) secondary antibody, then washed in TBST. Immunoblots were detected using ECL Prime or ECL Plus chemiluminescent reagent (GE Healthcare, Pittsburgh, PA, USA).

### uPA activity assay

uPA activity was measured in conditioned medium from the human CaP cell sublines LNCaP Neo/Snail, ARCaP Neo/Snail and 22Rv1 Neo/Snail using the uPA activity assay kit according to the manufacturer’s instructions. A chromogenic substrate is cleaved by active uPA to produce a colored product, which is detected on a plate reader at 405 nm. The concentration of active uPA was calculated relative to standards provided with the kit.

### siRNA transfection

Transient transfection of uPAR siRNA was performed on ARCaP Snail cells using DharmaFECT 1 reagent. Cells (1×10^6^/well) were seeded in a six-well plate and transfected with 200 nm uPAR-siRNA or control-siRNA in serum free media at 37°C with 5% CO_2_ for 5 h, followed by replacement of transfection media with RPMI-1640 supplemented with 5% DCC-FBS. After 72 h, transfected cells were harvested for western blot analysis of Snail, uPA, uPAR and β-actin; conditioned media was collected for the uPA activity assay. Transfected cells were also utilized for a subsequent invasion assay.

### Invasion assay

The invasive properties of the cell lines were measured using the BD BioCoat™ Matrigel™ Invasion guidelines. Briefly, Boyden chamber inserts (Thermo Fisher Scientific, Waltham, MA, USA) were coated with 50 μl 1:4 Matrigel and allowed to solidify at 37°C for 1 h. Cells were seeded in quadruplicate at 5×10^4^ (for ARCaP and 22Rv1) and 1×10^5^ (for LNCaP) in 0.1% FBS, while the lower chamber contained 10% FBS. Cells were treated accordingly and allowed to invade through the porous membrane coated with Matrigel at 37°C for 24–72 h. Inserts were fixed, stained and photographed in two fields per insert. Cell counts were performed for the determination of relative invasion or the stain solubilized with Sorenson solution and optical density measured at 590 nm.

### ERK inhibitor asssay treatments

The human CaP cell subline ARCaP Snail (1×10^6^), was cultured overnight. The following day, cells were treated with 20 μM ERK1/2 inhibitor (U0126) at the following time-points (0 and 30 min, 2, 6, 24 and 72 h). The conditioned media was collected and whole cell lysates were collected as previously described.

### Superarray analysis

Total RNA was isolated from ARCaP Neo or ARCaP Snail cells using the Qiagen kit according to the manufacturer’s instructions and 1 μg of which was reverse transcribed with oligo(dT) using MMLV-reverse transcriptase (Invitrogen Life Technologies, Carlsbad, CA, USA), to generate cDNA. The labeled cDNA was incubated with GEArray Q Series cancer pathway membranes (SuperArray, Valencia, CA, USA) at 60°C overnight. The membrane used in the present study contained 96 genes that were closely associated with cancer pathways, in addition to housekeeping control genes (such as GAPDH). After being washed, the membrane was incubated with streptavidin-alkaline phosphatase and was finally exposed to CDP-Star chemiluminescent substrate (SuperArray). Signal detection was performed using a high Performance chemiluminescence film (Amersham Biosciences, Amersham, UK). Analysis of results was performed using GEArray Expression Analysis Suite software (http://geasuite.superarray.com).

### Statistical analysis

All data are presented as the mean ± standard error of at least three independent experiments. The data were analyzed using two-way analysis of variance or Student’s t-test. All statistical analyses were performed and all graphs generated using GraphPad Prism 5.0 software (GraphPad Software Inc., San Diego, CA, USA). P<0.05 was considered to indicate statistically significant differences.

## Results

### Overexpression of Snail leads to an increase in cell invasion

**Figure 1:**
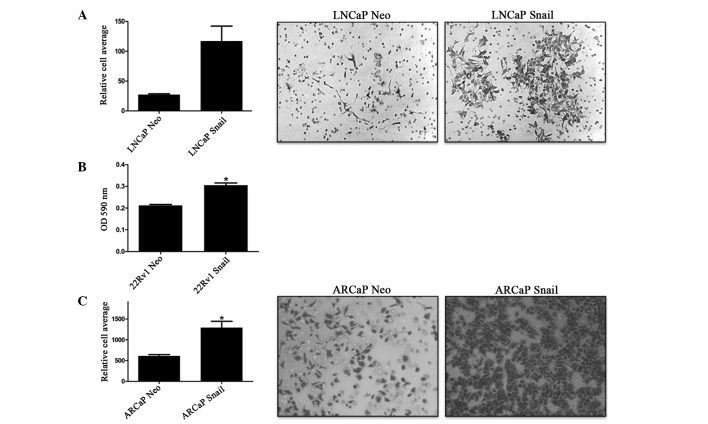
Snail overexpression increases cell invasion in prostate cancer cells. Prostate cancer cells lines, LNCaP, 22RV1 and ARCaP, stably transfected with empty vector (Neo) or constitutively active Snail cDNA using Lipofectamine 2000 were utilized for cell invasion assays using the Boyden chamber. (A) LNCaP Neo and Snail. Magnification, ×10. (B) 22RV1 Neo and Snail and (C) ARCaP Neo and Snail were plated at a density of 5×10^4^ cells in culture inserts coated with Matrigel. Magnification, ×10. Cells that invaded were stained with crystal violet and either counted or solubilized with Sorenson solution, and optical density (OD) was measured at 590 nm. These studies were performed in triplicate and the average number of cells from each repeat that invaded the Matrigel were deemed the ‘relative cell average.’ Results are representative of three independent experiments. Statistical analysis was performed using two-way analysis of variance or Student’s t-test (^*^P<0.05).

Previously, we have shown that Snail overexpression increases cell invasion in 22Rv1 cells (35). To confirm these results and examine the effect of Snail overexpression on LNCaP and ARCaP invasion through the ECM, an invasion assay was performed where Matrigel mimicked the ECM. As expected, Snail-transfected cells exhibited significantly more cell invasion compared with the Neo control-transfected cells in all three cell lines tested (Fig. 1). Therefore, Snail is associated with increased cell invasion.

### Overexpression of Snail leads to an upregulation of uPA and uPAR

**Figure 2:**
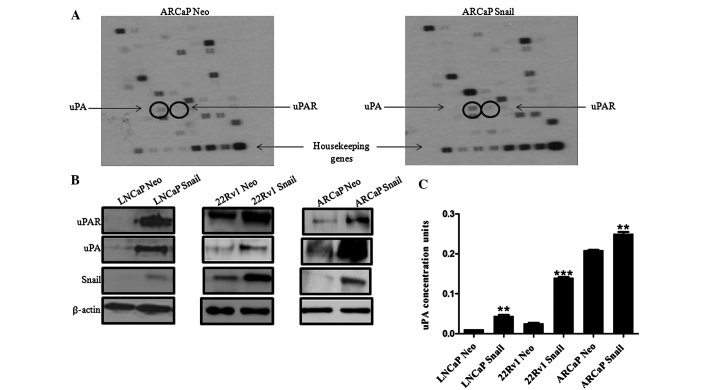
Snail increases urokinase-type plasminogen activator (uPA)/uPA receptor (uPAR) levels and uPA activity in prostate cancer cells. (A) Total RNA was isolated from ARCaP empty vector (Neo) or ARCaP Snail cells. Superarray analysis was performed utilizing gene expression arrays for cancer pathways. uPA and uPAR RNA levels were upregulated by Snail. (B) Snail, uPA and uPAR protein expression was determined by western blot analysis. Actin was utilized as the loading control. (C) Secreted uPA activity was measured in conditioned media using uPA activity assay. Results are representative of three independent experiments. Statistical analysis was performed using two-way analysis of variance or Student’s t-test (^**^P<0.01 and ^***^P<0.001).

In order to examine the molecular mechanism by which Snail may increase cell invasion, a superarray analysis was performed on ARCaP Neo and ARCaP Snail CaP cells to identify genes downstream of Snail that may be responsible for the increase in cell invasion. Notably, a protein associated with cell invasion, uPA, and its receptor, uPAR, were upregulated (Fig. 2A). Subsequently, the protein expression levels of uPAR and its ligand uPA were evaluated in Snail overexpressing LNCaP, 22Rv1 and ARCaP cells. In all three CaP lines, Snail transfection increased uPA and uPAR protein expression (Fig. 2B). Additionally, measurement of secreted uPA activity in conditioned media showed that LNCaP, 22Rv1 and ARCaP cell lines overexpressing Snail exhibited higher uPA activity compared with that of the Neo control (Fig. 2C). The results also suggested that the androgen-independent ARCaP cells had a higher active uPA concentration compared with that of the androgen-dependent LNCaP and 22Rv1 cells. Therefore, Snail is associated with increased uPa/uPAR protein levels and increased uPA activity.

### uPAR knockdown in Snail-overexpressing ARCaP cells leads to decreased cell invasion

**Figure 3:**
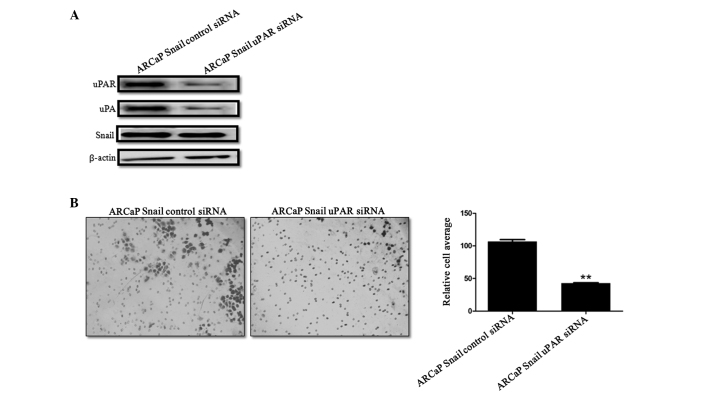
Snail mediates cell invasion via urokinase-type plasminogen activator receptor (uPAR). Transient transfection of control or uPAR short interfering RNA (siRNA) was performed on ARCaP Snail cells using DharmaFECT 1 reagent. (A) uPA, uPAR and Snail expression was determined by western blot analysis. Actin was utilized as the loading control. (B) Invasion through Matrigel was tested on the cells treated with control siRNA and uPAR siRNA using the Boyden chamber. Results are representative of three independent experiments. Cells were stained with crystal violet and rinsed in distilled H_2_O. Magnification, ×10. These studies were performed in triplicate and the average number of cells from each repeat that invaded the Matrigel were deemed the ‘relative cell average.’ Statistical analysis was performed using two-way analysis of variance or Student’s t-test (^**^P<0.01).

To evaluate the contribution of uPA/uPAR in the increased invasion that was observed in the Snail overexpressed cells, uPAR was transiently knocked down in ARCaP Snail cells. Western blot analysis confirmed the knockdown of uPAR (Fig. 3A). Of note, uPAR knockdown was accompanied by a decrease in uPA expression, while Snail expression was not affected by this knockdown (Fig. 3A). Functionally, there was a significant decrease in invasion following uPAR knockdown (Fig. 3B). Thus, uPAR contributes to Snail-mediated cell invasion.

### Inhibition of MAPK activity downregulates uPA activity and decreases cell invasion

**Figure 4:**
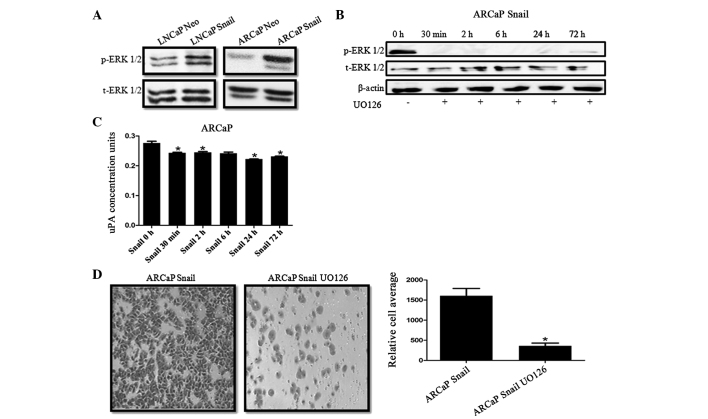
Extracellular-signal-regulated kinase (ERK) inhibition antagonizes Snail-mediated urokinase-type plasminogen activator (uPA) activity and cell invasion. (A) Phospho-ERK1/2 (p-ERK), total ERK1/2 (t-ERK) and Snail expression were determined by western blot analysis in LNCaP and ARCaP cells overexpressing Snail. Actin was utilized as the loading control. (B) ARCaP Snail cells were treated with UO126 MEK inhibitor for various time periods. (C) Secreted active uPA activity was measured in conditioned media using uPA activity assay. (D) Invasion through Matrigel was tested on ARCaP Snail cells without and with UO126 for 24 h using the Boyden chamber. Cells were stained with crystal violet and rinsed in distilled H_2_O. Magnification, ×10. These studies were performed in triplicate and the average number of cells from each repeat that invaded the Matrigel were deemed the ‘relative cell average.’ Results are representative of three independent experiments. Statistical analysis was performed using two-way analysis of variance or Student’s t-test (^*^P<0.05).

We have previously demonstrated that there is an increase of phosphorylated MAPK (p-ERK) in CaP cells overexpressing Snail (34,36). Therefore, we investigated whether Snail regulation of uPA activity was mediated by MAPK signaling. It was identified that Snail overexpression increases ERK activity in LNCaP and ARCaP cell lines (Fig. 4A). Subsequently, Snail-transfected ARCaP cells were treated with 20 μM UO126 MEK inhibitor for 30 min and 2, 6, 24 and 72 h. Decreased ERK activity was observed by 30 min and persisted until 72 h as shown by the western blot analysis (Fig. 4B). It was also revealed that inhibiting MAPK activity significantly decreased uPA activity within 30 min (Fig. 4C). Finally, ARCaP Snail cells treated with U1O26 for 24 h showed decreased invasive potential compared with that of the ARCaP Neo control (Fig. 4D).

## Discussion

Studies have suggested that epithelial mesenchymal transition (EMT) is an important step leading to cancer metastasis (7–9). One mechanism by which E-cadherin is downregulated in EMT is transcriptional repression by Snail (10,11). In the present study, we have shown that overexpression of Snail increases cell invasion in androgen-dependent LNCaP and 22RV1 prostate cancer cell lines and androgen-independent ARCaP prostate cancer cell lines. In Snail-transfected ARCaP cells, certain genes that were upregulated and downregulated were evaluated via superarray analysis, based on their function. The results of the superarray demonstrated that the overexpression of Snail leads to upregulation of genes involved with invasion and metastasis, such as uPA and uPAR. It was noteworthy that uPA and uPAR were upregulated in Snail-transfected CaP cells, as in previous studies performed in PC3 and DU145 cells, RNA interference of uPA and uPAR resulted in uPA and uPAR mRNA and protein expression being completely inhibited and there was a decline in metastasis (38). Although the signaling cascade resulting in the expression of uPA and uPAR being downregulated was not determined, the superarray analysis and uPAR siRNA studies done in Snail-transfected cells suggest that it may be through Snail. To confirm our superarray studies, we showed that uPA and uPAR protein expression was increased in Snail-overexpressing cells. Additionally, Snail overexpression led to increased uPA activity. Although there was a general increase in uPA activity in the Snail-transfected cells, there was a greater level of uPA activity in the androgen-independent ARCaP cells compared with that in the androgen-dependent LNCaP and 22Rv1 cells.

To determine the effect the uPA/uPAR system has on the increase in invasion in Snail-transfected cells, uPAR was transiently knocked down. The most well known activator of uPA is uPAR; therefore; knocking down uPAR inhibits the function of both uPA and uPAR (19). We observed that uPAR knockdown in ARCaP Snail cells led to a significant decrease in cell invasion. It is noteworthy that Snail expression was not affected by the knockdown of uPAR, suggesting that uPAR is acting downstream of Snail to increase cell invasion; thus, for the first time, we show that Snail relies on uPAR to increase invasion. It may be suggested that uPA/uPAR signaling alone does not have an important role in Snail-mediated invasion in ARCaP cells, as uPAR knockdown did not completely eliminate invasion. Previously, we have shown that ERK activity is increased in Snail-transfected ARCaP cells (34,36). In the present study, in order to determine whether Snail mediates invasion through the MAPK pathway, Snail-transfected cells were treated with MEK inhibitor UO126 for various time periods. uPA activity and invasion was significantly decreased in ARCaP Snail cells treated with UO126 in a time-dependent manner. This suggests that Snail may use the MAPK pathway to mediate cell invasion through uPA/uPAR signaling in ARCaP cells. Supporting these results, the literature suggest that uPAR is under an ERK-dependent mechanism and blocking uPAR’s activity leads to inhibition of motility in hepatocellular carcinoma (27). Additionally, a study on human gastric cancer has shown that EGF stimulates uPAR expression via the ERK pathway, sequentially increasing cell invasion (28). Although the activity of uPA was decreased upon MAPK inhibition, it was not completely eliminated, possibly since its activity may be mediated by additional pathways, such as AKT. In breast cancer, studies have shown that upon uPA binding to uPAR, AKT is activated (39,40).

Overall, the present results show, for the first time, a link between Snail, MAPK and uPA/uPAR in CaP. Our studies suggest that Snail overexpression increases cell invasion through the upregulation of uPA/uPAR signaling, which is mediated in part by the MAPK signaling pathway.

## Abbreviations

EMT: epithelial to mesenchymal transition
uPA: urokinase plasminogen activator
uPAR: urokinase plasminogen activator receptor
CaP: prostate cancer
MAPK: mitogen-activated protein kinase
